# A Novel and Minimally Invasive Approach Using the Root and Cervical Margin Flattening Procedure for Treating Gingival Recession: A Report of Four Cases

**DOI:** 10.7759/cureus.65142

**Published:** 2024-07-22

**Authors:** Kazunari Ando, Daiki Ando, Yuki Kojima

**Affiliations:** 1 Dentistry, Kazu Dental Clinic, Kamisu, JPN; 2 Anesthesiology, Asahi General Hospital, Asahi, JPN

**Keywords:** dentistry, gingivoplasty, root planing, gingival recession, periodontal diseases

## Abstract

Traditional plastic surgery techniques for root coverage using connective tissue grafts are often invasive and cause patient discomfort. A new procedure with a minimally invasive approach for gingival recession was developed and termed the “root and cervical margin flattening procedure.” A blunt incision was performed in the buccal gingival sulcus at the alveolar bone crest with a dissector or raspatory. After the incision, a split-thickness flap was dissected extending beyond the mucogingival junction, palpating the alveolar bone crest with a periodontal probe and flattening the cervical region and roots to smooth out irregularities along the dental root. In some complicated cases, more reliable effects were expected using a periodontal tissue regeneration drug and protective splint. The creeping attachment distance reached the flattened area. Careful blood clot preservation was crucial in the postoperative period. The gingival creeping attachment implied two main factors. First, surgical invasion could promote healing. Second, soft tissue space was increased due to root flattening. This simple and minimally invasive approach for treating cervical lesions (including non-carious cervical lesions and cervical/root caries) and gingival recession could obviate the need for connective tissue grafts. Further clinical studies are required to assess its success and prognosis.

## Introduction

Aging populations and life expectancies are increasing worldwide. By 2030, one in six people worldwide will be aged ≥ 60 years. The demographic of those aged ≥ 60 years is set to increase from one billion in 2020 to 1.4 billion and will double in 2050 [1]. Owing to advances in dental care and increased dental awareness, tooth loss has significantly declined in later life [2]. However, gingival recession is induced by aging, periodontitis, and inadequate brushing [3]. Approximately half the gingival recession cases are associated with non-carious cervical lesions (NCCL) [4]. In addition, almost half the older adults are affected by dental caries, particularly cervical and root caries [5,6]. Dental restorations addressing cervical and root caries may influence gingival recession [7]. This is because the position of the restorative margin can interfere with connective tissue attachment. Furthermore, subgingival restoration margins may lead to inflammation because unpolished or overhanging surfaces tend to accumulate more plaque than smooth surfaces [7].

A variety of common treatments of gingival recession exist, including tooth brushing instructions, scaling, root planing, use of coronally advanced flaps and connective tissue grafts, and application of citric acid/ethylenediaminetetraacetic acid/enamel matrix derivatives [3]. The traditional plastic surgery techniques for root coverage using connective tissue grafts are associated with increased patient morbidity and discomfort [8]. Therefore, treating cervical lesions and gingival recession is often difficult and complex. Núñez et al. [9] and Romano et al. [10] reported that reduced avascular root surfaces permit increased gingival margin thickness and creeping via modified root surfaces. Diminishing root convexity enables gingival thickness augmentation. This provides a concave space for the formation and stability of a blood clot, eventually leading to increased tissue thickness [10]. These reports indicated that root flattening could induce gingival creeping. To combine this process and blunt dissection technique, we developed a more effective and minimally invasive approach for gingival recession compared with conventional methods.

## Case presentation

Root and cervical margin flattening procedure

**Figure 1:**
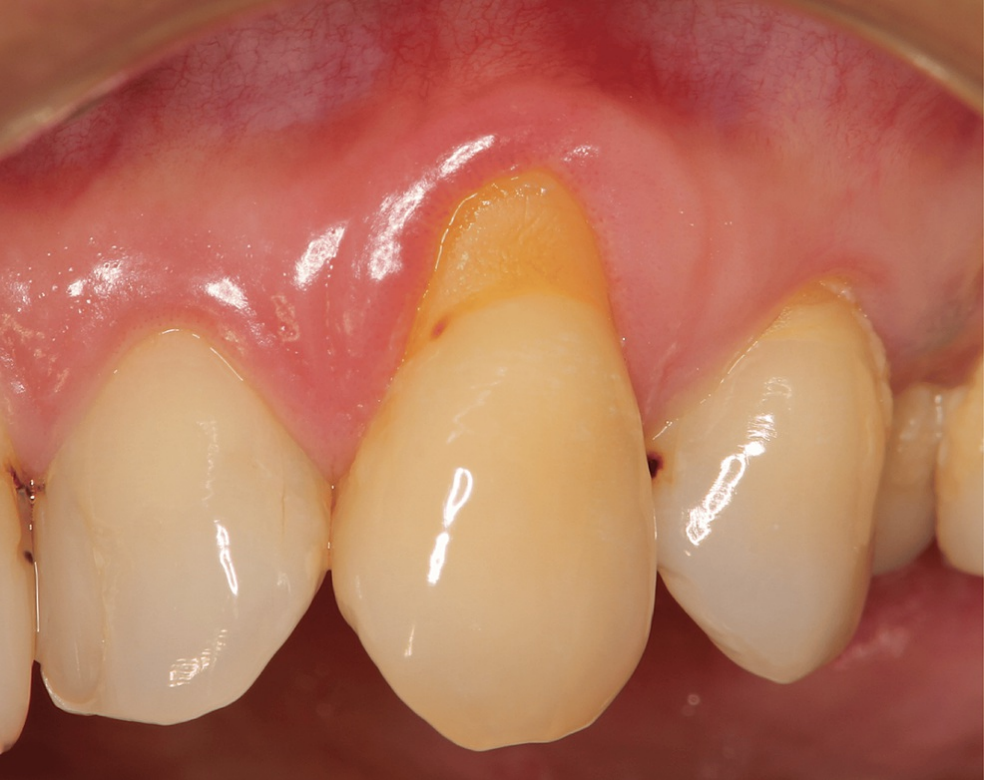
Preoperative image in Case 1 Gums are inflammation-free, plaque control is good, and periodontal pockets are ≦ 3 mm.

The surgical indications for this technique were as follows: Cairo’s classification of gingival recession defects-type 1 or 2 [11], presence of healthy periodontal tissue, absence of clinical signs of inflammation, probing depth within 3 mm, and no bleeding on probing. Cone beam computed tomography (CBCT) can provide informative radiographic findings and is applicable to all types of cervical abrasions. All four patients in this report were informed of the possibility of gingival recession and NCCL progression due to improper brushing and were instructed on the appropriate brushing techniques. Preoperative preparations aimed to achieve plaque control record and bleeding on probing of 20% or less (Figure 1).

**Figure 2:**
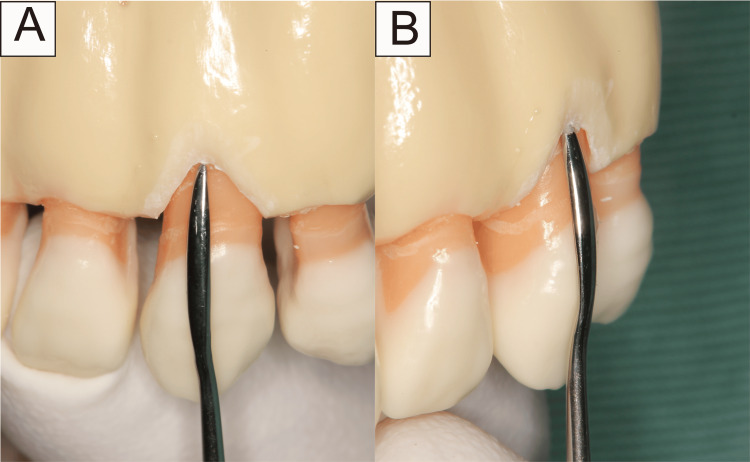
Cervical incision line in model (A) front view; (B) side view. The dissection is performed within the buccal gingival sulcus, reaching the alveolar bone crest. When dissecting beyond the alveolar bone crest, the dissector or raspatory should be turned to the opposite side.

**Figure 3:**
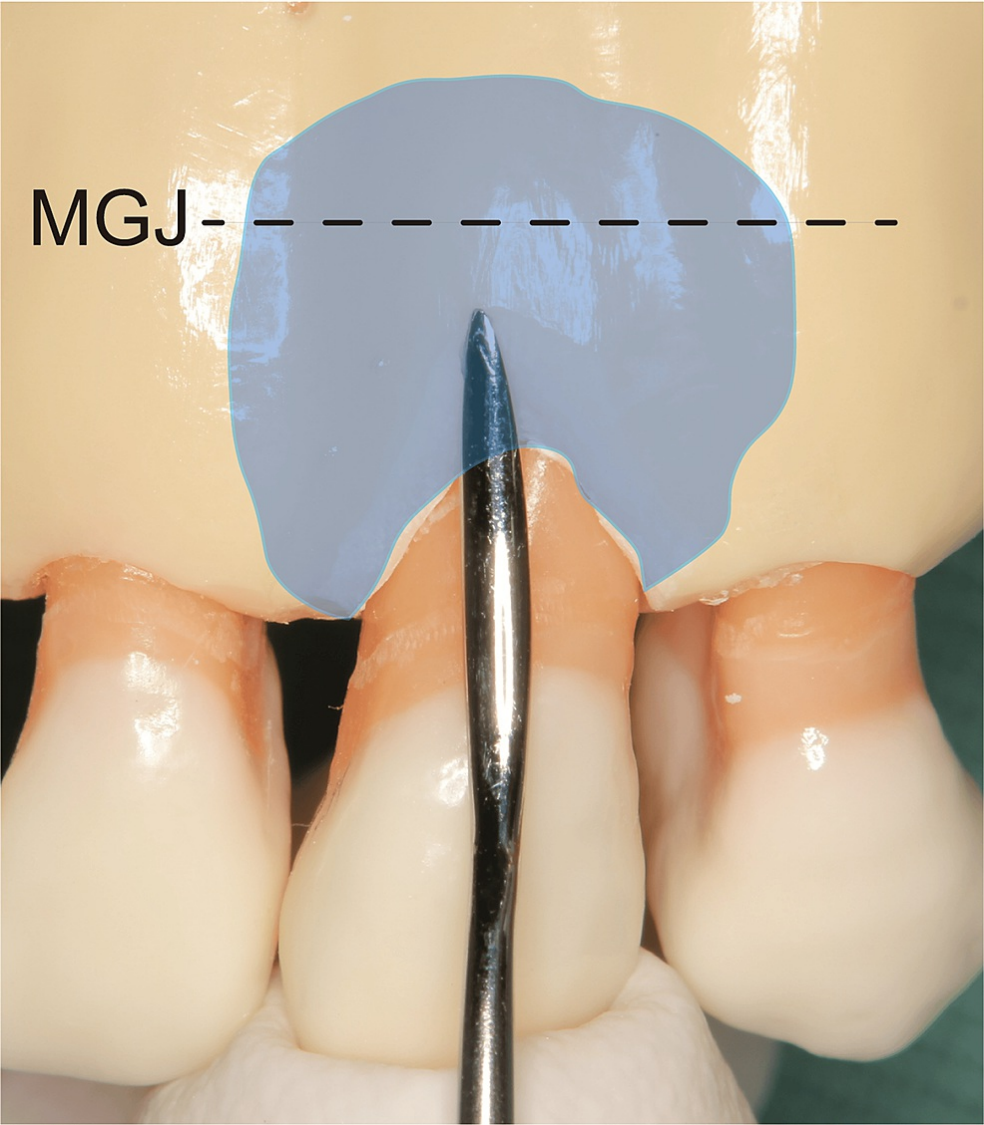
Blunt dissection in tooth model The area of peeling is carried out beyond the mucogingival junction. The flap is carefully removed to avoid creating a hole in the flap and create a partial-thickness flap. The split-thickness flap is carefully formed by blunt dissection extending beyond the mucogingival junction as an envelope flap, while avoiding perforation. MGJ: mucogingival junction

**Figure 4:**
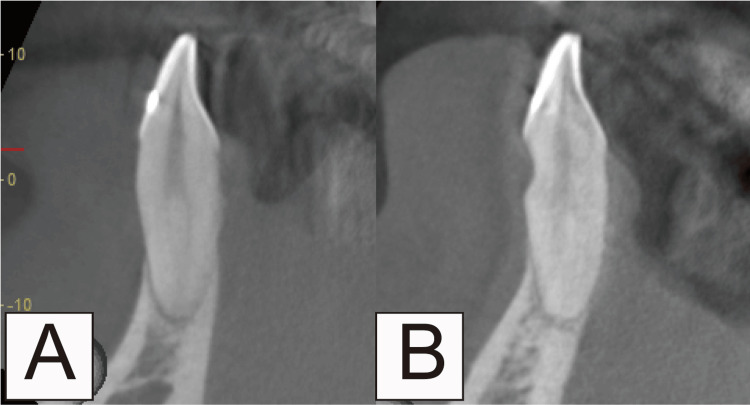
Flattening in a case of cervical dentin defect shown by cone beam computed tomography Cutting is performed to ensure the missing tooth structure and root flattening area are smooth. If there is no tooth loss in the cervical region, the tooth is designed and cut to be smooth in the area where root flattening is performed. In cases wherein the root shape is deformed, or the tooth is tilted and flattening is difficult to determine, a cone beam computed tomography scan should be performed. (A) Before root and cervical margin flattening procedure; (B) After root and cervical margin flattening procedure.

Following confirmation of local anesthesia application to the relevant area, a blunt incision was made from the buccal gingival sulcus to the alveolar bone crest using a dissector or raspatory (Figure 2). Subsequently, a split-thickness flap was carefully dissected, bluntly extending beyond the mucogingival junction (MGJ) as an envelope flap, while avoiding perforation (Figure 3). Only by dissecting to the MGJ, the gingiva could be confirmed as coronally moved. Furthermore, the movable gingival flap reduced the soft tissue damage caused by the rotary cutting instrument. Flattening was performed after confirming that the dissected area formed a sector. The scope of flattening could be categorized into two groups. If there was a dentin defect in the cervical region, this area was also included in the flattened area. Root flattening was performed in cases of no cervical lesion to smooth the tooth in that area. With this technique, CBCT scanning was not necessary. However, in cases wherein the root shape was deformed, or the tooth was tilted and flattening was difficult to determine, a CBCT scan was performed. (Figure 4).

**Figure 5:**
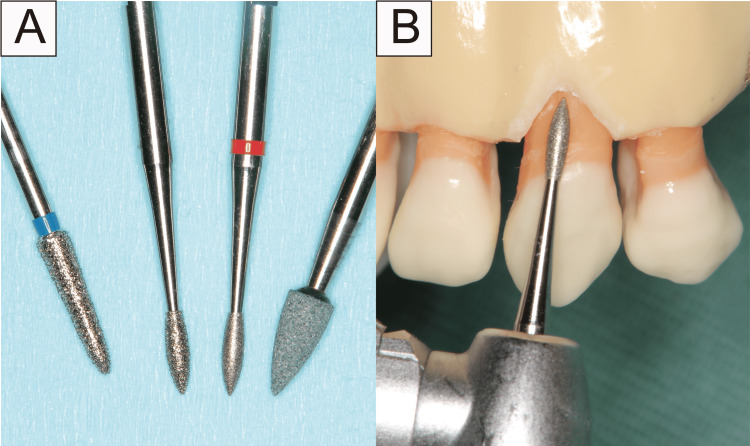
Flattening technique in tooth model This is done using a diamond bur or root planning bur.

There are two points to consider when flattening a tooth root: (i) determining the horizontal depth of the area to be dissected and (ii) palpating the alveolar bone margin with a probe and smoothening out the unevenness of the dental root between the MGJ and cervical region. The second step was to smooth the flattened root surface and crown using a dental rotary instrument with a diamond or root planing bur (Figure 5). Therefore, this flattening procedure comprised odontoplasty of the tooth. No suturing of the dissected gingiva area was performed. This technique could apply the healing mechanism of surgical invasion.

**Figure 6:**
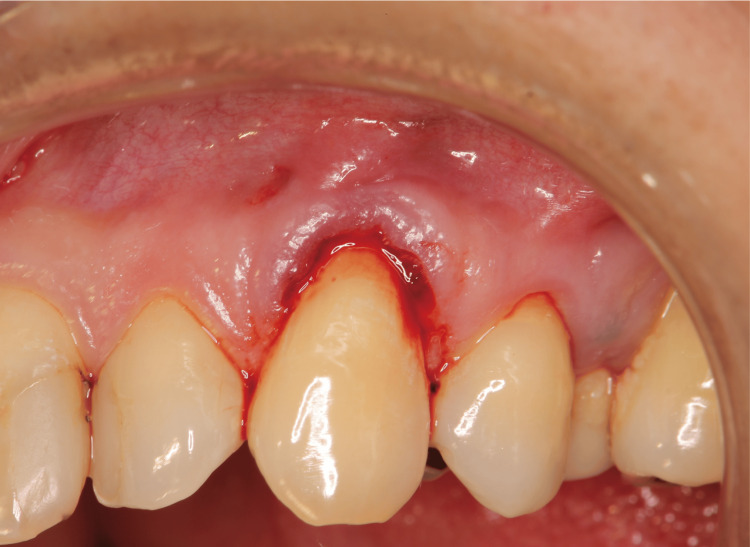
Blood clots during root and cervical margin flattening procedure in Case 1 This technique applies the healing mechanism of blunt dissection. The most crucial factor in the healing process is to preserve blood clots.

**Figure 7:**
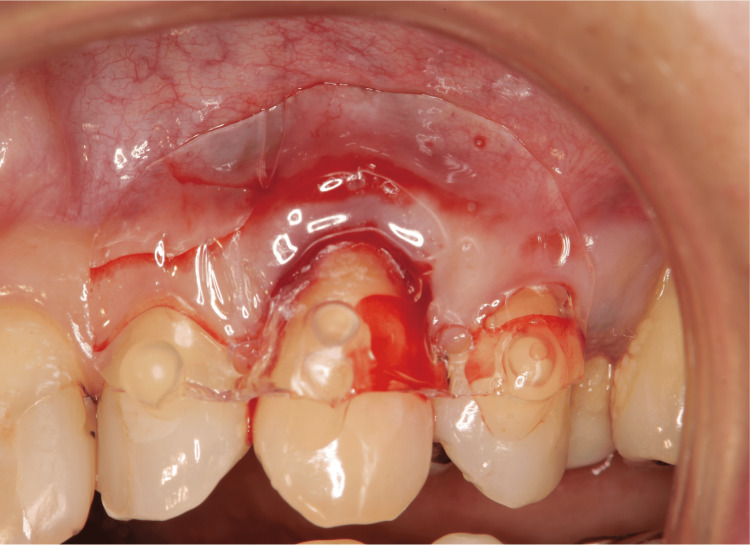
Combined with periodontal tissue regeneration drug and protective splint in Case 1 If there is a high risk of disruption of blood clots falling off, the formation of which is an essential factor in healing, a more reliable effect can be expected using periodontal tissue regeneration drugs and protective splints.

The most crucial factor in the healing process was preserving the blood clots (Figure 6). Diabetes mellitus or other systemic diseases might impair wound healing. In such cases, a more reliable effect could be expected using a periodontal tissue regeneration drug (Regroth; KAKEN Pharmaceutical Co., Ltd., Japan) and a protective splint (Splint disc 1 mm; Vital-net Inc., Japan) (Figure 7). The protective splint was composed of polyethylene terephthalate. The periodontal tissue regeneration drug was applied below the gingival flap to the cervical line.

**Figure 8:**
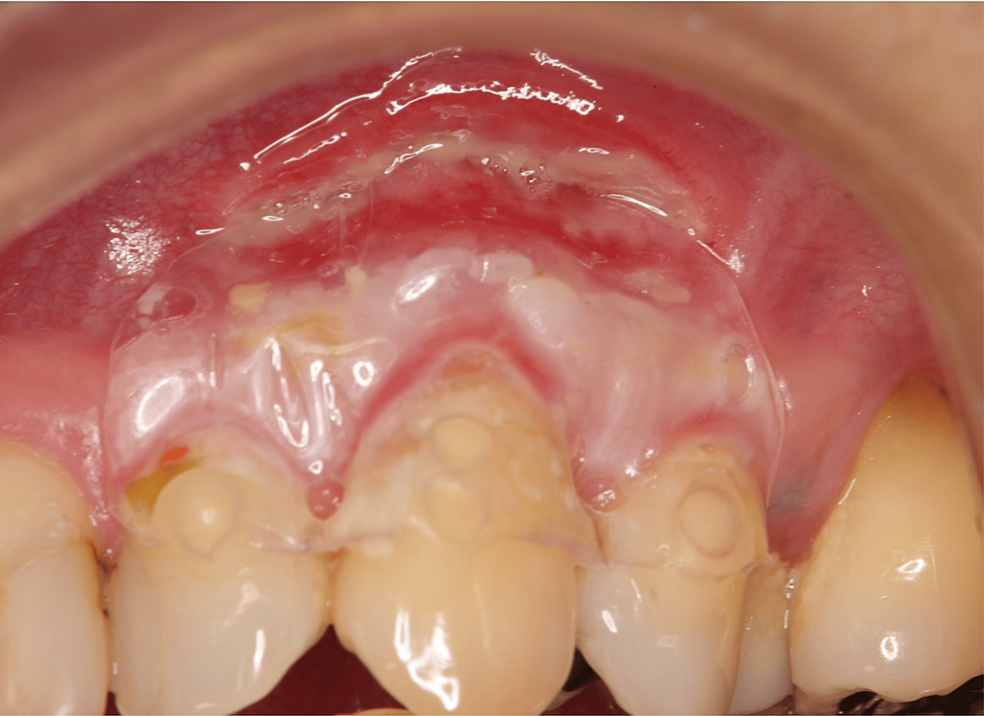
Postoperative image after one week in Case 1 The splint is removed, and the wound is checked.

**Figure 9:**
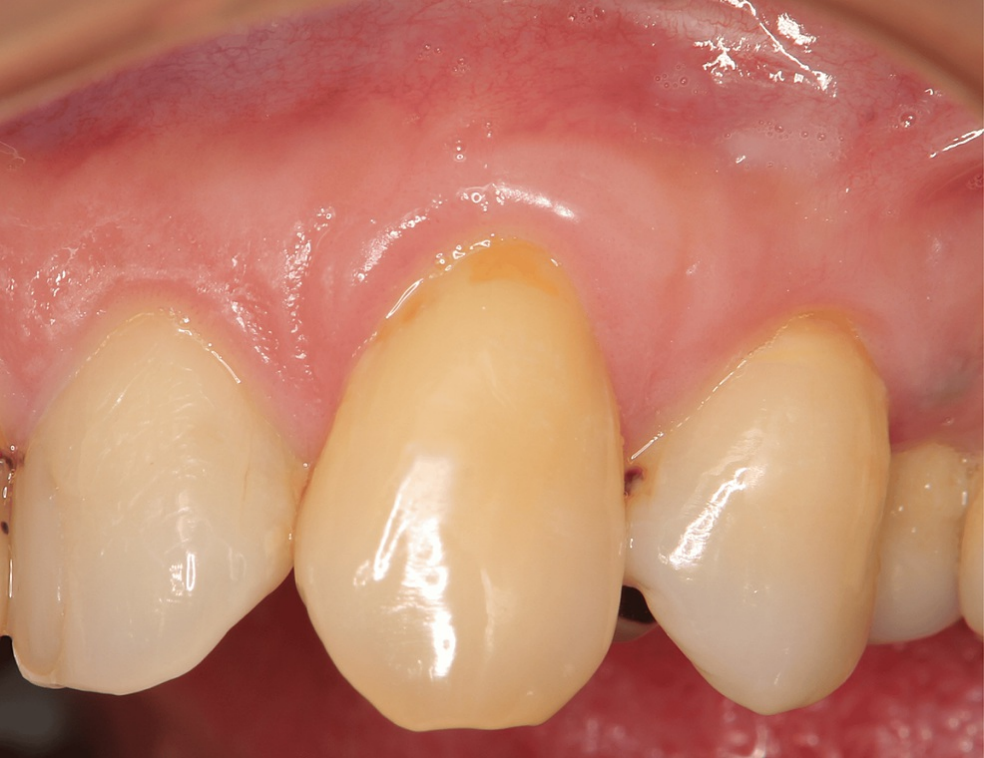
Postoperative image after 12 months in Case 1

The protective splint was removed one week post surgery (Figure 8). The following points were explained to the patients: Avoidance of brushing the surgical area for a week and the symptoms of dentin hypersensitivity that may occur. The healing process was observed for 12 months when the creeping distance reached its maximum. Creeping typically extended to the flattened area (Figure 9). Postoperative management included caution against premature removal of blood clots. We termed this technique as the “root and cervical margin flattening procedure” (RCFP). The most unique characteristic of the RCFP was to dissect bluntly extending beyond the MGJ. By forming this envelope flap, the gingiva moved coronally and promoted good creeping. This technique was simpler and less invasive compared with conventional coronally advanced flaps and connective tissue grafts.

Case 1

The patient, a 54-year-old woman with type 2 diabetes mellitus, presented to our clinic primarily with gingival recession (Figure 1). According to Cairo’s classification, this case was categorized as RT1. Treatment involved administration of periodontal tissue regeneration drugs and use of a protective splint (Figures 6, 7). After 12 months, the creeping distance was 2.30 mm (Figure 9).

Case 2

**Figure 10:**
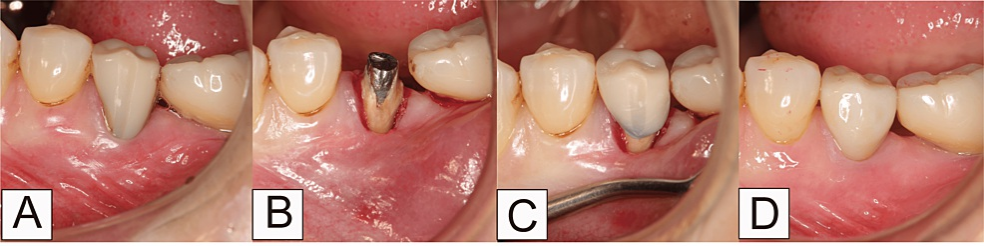
Thin keratinized gingiva in the mandibular right molar area in Case 2 (A) Before root and cervical margin flattening procedure (RCFP); (B) During RCFP; (C) The temporary crown is set; (D) 12 months after RCFP

The patient, a 64-year-old woman, presented to our clinic reporting pain while brushing her lower left molars. There was little keratinized gingiva in the mandibular right molar area (Figure 10A). According to Cairo’s classification, this case was categorized as RT2. The RCFP was performed after removing the long resin jacket crown (Figure 10B). In this case, periodontal tissue regeneration drugs and a protective splint were not used. Performing the RCFP, a crown with a shortened tooth crown was set (Figure 10C). After 12 months, the creeping distance was 2.14 mm; creeping led to more keratinized gingiva, which made brushing easier. The width of the keratinized tissue increased by 1.56 mm (Figure 10D).

Case 3

**Figure 11:**
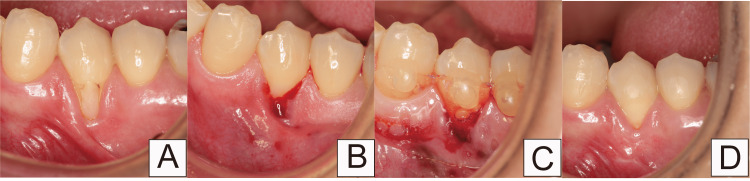
After filling the composite resin to the cervical region in Case 3 (A) Before root and cervical margin flattening procedure (RCFP); (B) During RCFP; (C) The splint is set with a periodontal tissue regeneration drug; (D) 12 months after RCFP

The patient, a 54-year-old woman, presented to our clinic, mainly with gingival recession. After restoring the cervical region with composite resin, the gingival recession was attributed to inappropriate plaque control (Figure 11A). According to Cairo’s classification, this case was categorized as RT1. The composite resin was removed; the RCFP was performed (Figure 11B). A periodontal tissue regeneration drug and protective splint were combined to preserve the blood clot (Figure 11C). The splint was removed after one week. The creeping distance was 2.10 mm over 12 months. The width of keratinized tissue increased by 1.72 mm (Figure 11D).

Case 4

**Figure 12:**
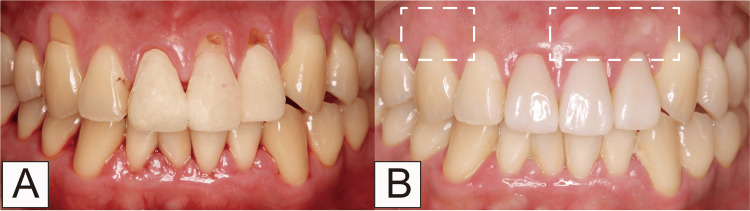
Comparison of root and cervical margin flattening procedure with connected tissue grafts in Case 4 (A) Initial visit; (B) 12 months after the procedure

The patient, a 28-year-old man, visited our clinic reporting gingival recession on 13, 21, 22, and 23. Additionally, root carriers existed on 21 and 22 (Figure 12A). According to Cairo’s classification, this case was categorized as RT1. RCFP was performed on 13, 21, 22, and 23. On 21 and 22, when complete root coverage could not be achieved, gingival flap coronal movement and connective tissue graft were performed. Examination, 12 months later, revealed that scar tissue was found on the transplanted on 21 and 22. No scar tissue was observed on 13 and 23 (Figure 12B). The creeping distance was 2.94 mm; the keratinized tissue width increased by 1.02 mm.

## Discussion

We presented a novel surgical approach for treating cervical lesions and gingival recession, distinct from the traditional methods, by omitting connective tissue graft procedures. We anticipate its applicability across various regions. The most unique characteristic of the RCFP is dissection bluntly extending beyond the MGJ. By forming this envelope flap, the gingiva moved coronally and promoted good creeping. This technique was simpler and less invasive compared with conventional coronally advanced flaps and connective tissue grafts. The RCFP is a highly novel technique. In these four cases, we were unable to examine the percentage of root coverage because the cement enamel junction was not clear. Presently, we evaluated the amount of creeping and the width of the keratinized gingiva based on the relative distance between the gingival margin and the MGJ and the coronal incisal edge. In the future, conducting research involving the determination of root coverage percentage is necessary.

In general, the mechanisms of creeping attachment involve several factors. The contractile properties of fibroblasts may induce attachment creeping [12]. Additionally, the overhealing process progressively covers denuded surfaces with proliferating cells of newly formed gingival tissue [12,13]. Ando et al. reported improvements in multiple gingival recessions through non-surgical and supportive periodontal therapy, such as root flattening [14,15].

Possible reasons for creeping following the RCFP could include surgical invasion inducing healing, soft tissue space augmentation through root flattening, and valve horizontal and vertical movements due to avulsion beyond the MGJ. Hence, anticipating a robust healing mechanism is essential for RCFP success. The RCFP with an enamel matrix derivative or a periodontal tissue regeneration drug might be an effective treatment in patients prone to healing failure due to diabetes mellitus or malnutrition, as several reviews have concluded that an enamel matrix derivative improves root coverage compared with surgical procedures alone [16,17]. Additionally, if gingival recession cannot be improved using the RCFP, a coronally advanced flap with connective tissue grafts should be considered. Núñez et al. reported a two-step surgical approach with flattening of the root surface that was effective in gingival recession [9]. Cases in which RCFP should not be performed include uncontrolled diabetes mellitus or other systemic diseases, deep periodontal pockets or inflammation, extremely thin gingival phenotype, severe dentinal hypersensitivity, and deep caries or deep non-carious cervical lesions.

Complications of the RCFP can occur. One patient developed subcutaneous emphysema. This was due to using a rotary cutting instrument under the mucous membrane, which might have caused air to enter the subcutaneous area [18]. If subcutaneous emphysema occurs, patient monitoring and antibiotic administration if symptoms occur are necessary. Another complication can be mental nerve palsy as dental surgery on the mandibular premolar region may damage the mental nerve [19]. Finally, the onset and worsening of temporary hyperesthesia occurred post surgery, but the symptoms disappeared after coverage was achieved. CBCT can confirm the mental foramen position, averting such issues. However, further clinical investigations and statistical analysis of success rates and indications are warranted.

## Conclusions

This report describes a simple and minimally invasive approach to treat cervical lesions and gingival recession. This technique does not require connective tissue grafts. However, there are limitations to its applicability. If clinical research is conducted using this technique in the future, more effective treatment could be developed. Therefore, applying it in many cases might be possible. Further clinical studies are required to evaluate this technique's success rates rand long-term prognosis.
